# Supplementary figures and images for: Cryptic Genes for Interbacterial Antagonism Distinguish Rickettsia Species Infecting Blacklegged Ticks From Other Rickettsia Pathogens
Source: Front Cell Infect Microbiol. 2022 May 3;12:880813. doi: 10.3389/fcimb.2022.880813 (PMC9111745; doi:10.3389/fcimb.2022.880813)

A

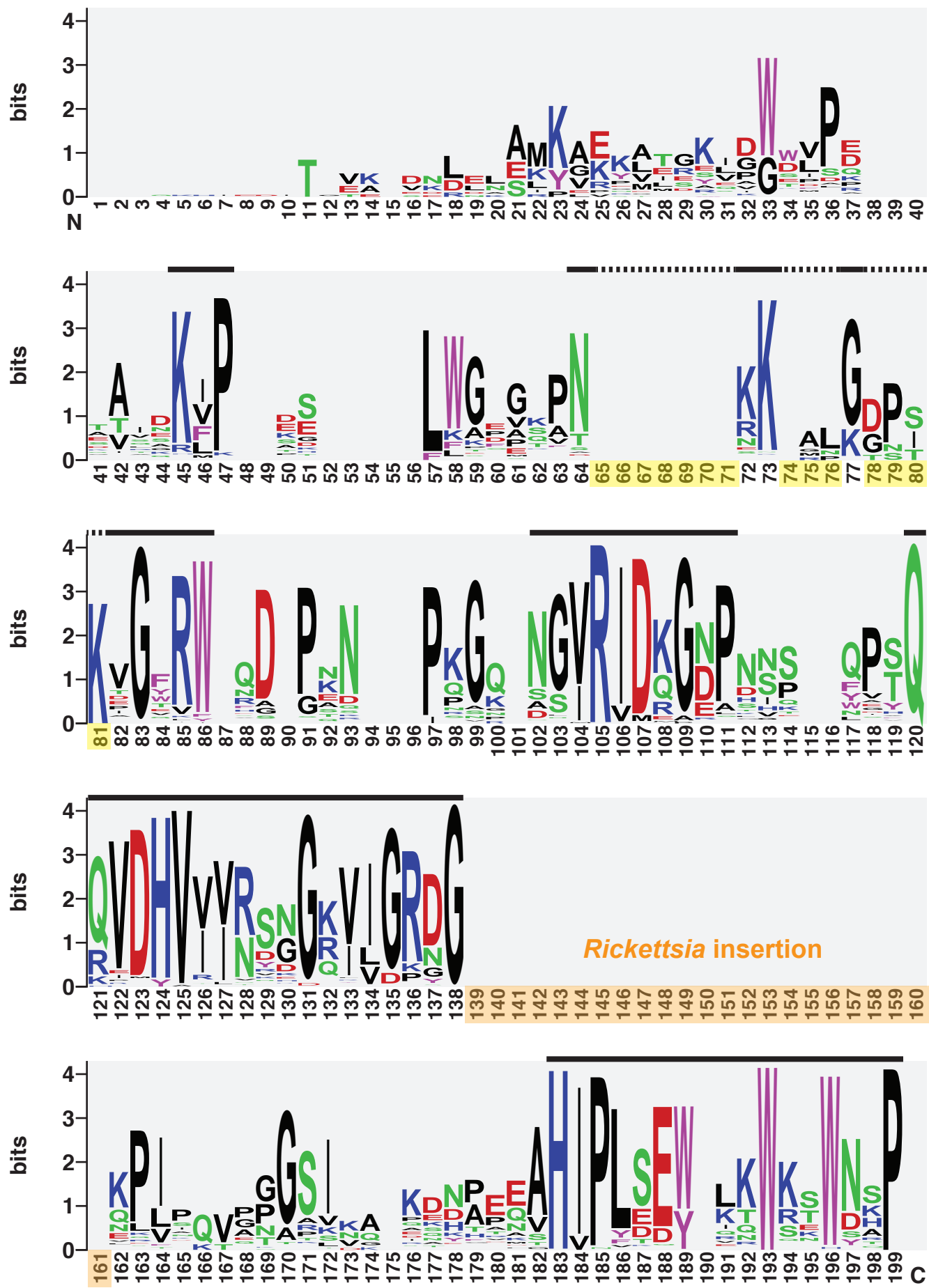

Fig. S1

Supplement: Supplementary Figure 1 — Sequence analysis of 155 predicted CRCTs integrated into diverse bacterial toxins. (A) Sequence logos (Crooks et al., 2004) depict complete protein alignment generated using MUSCLE, default parameters (Edgar, 2004). Information for all proteins is provided in Table S1 . Regions shown in Figures 2 are denoted with a bar above the logos. Amino acid coloring as follows: black, hydrophobic; red, negatively charged; green, hydrophilic; purple, aromatic; blue, positively charged. Unique Rickettsia insertion is shown in orange. Other insertions noted by triangles in Figure 2 are highlighted yellow). (B) HaloBlast profiles for REIS_1424 with and without a 23 aa insertion (see Figure 2E and text for more details). [file DataSheet_1.pdf]

B

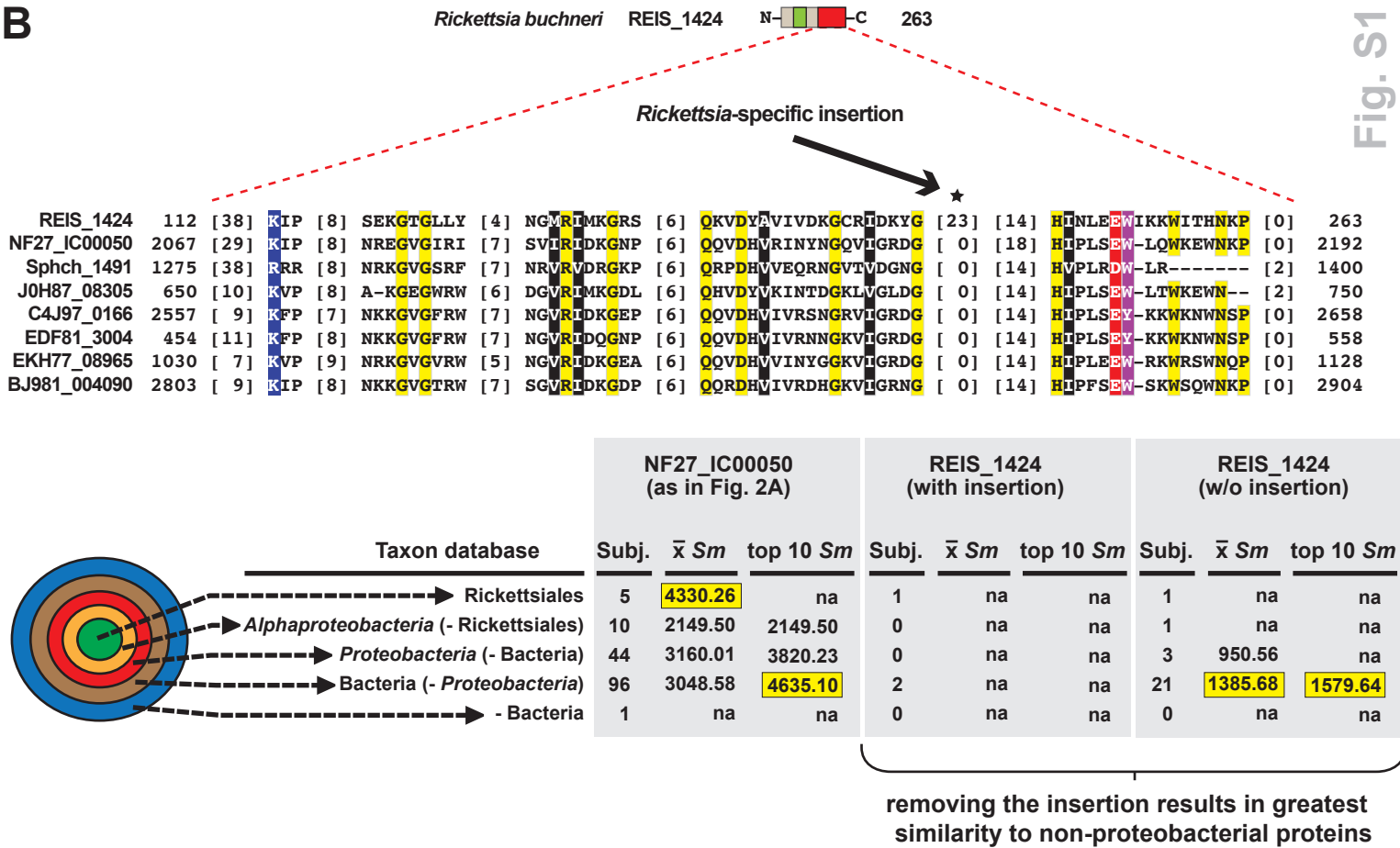

Fig. S1

Supplement: Supplementary file 2 [file DataSheet_2.pdf]

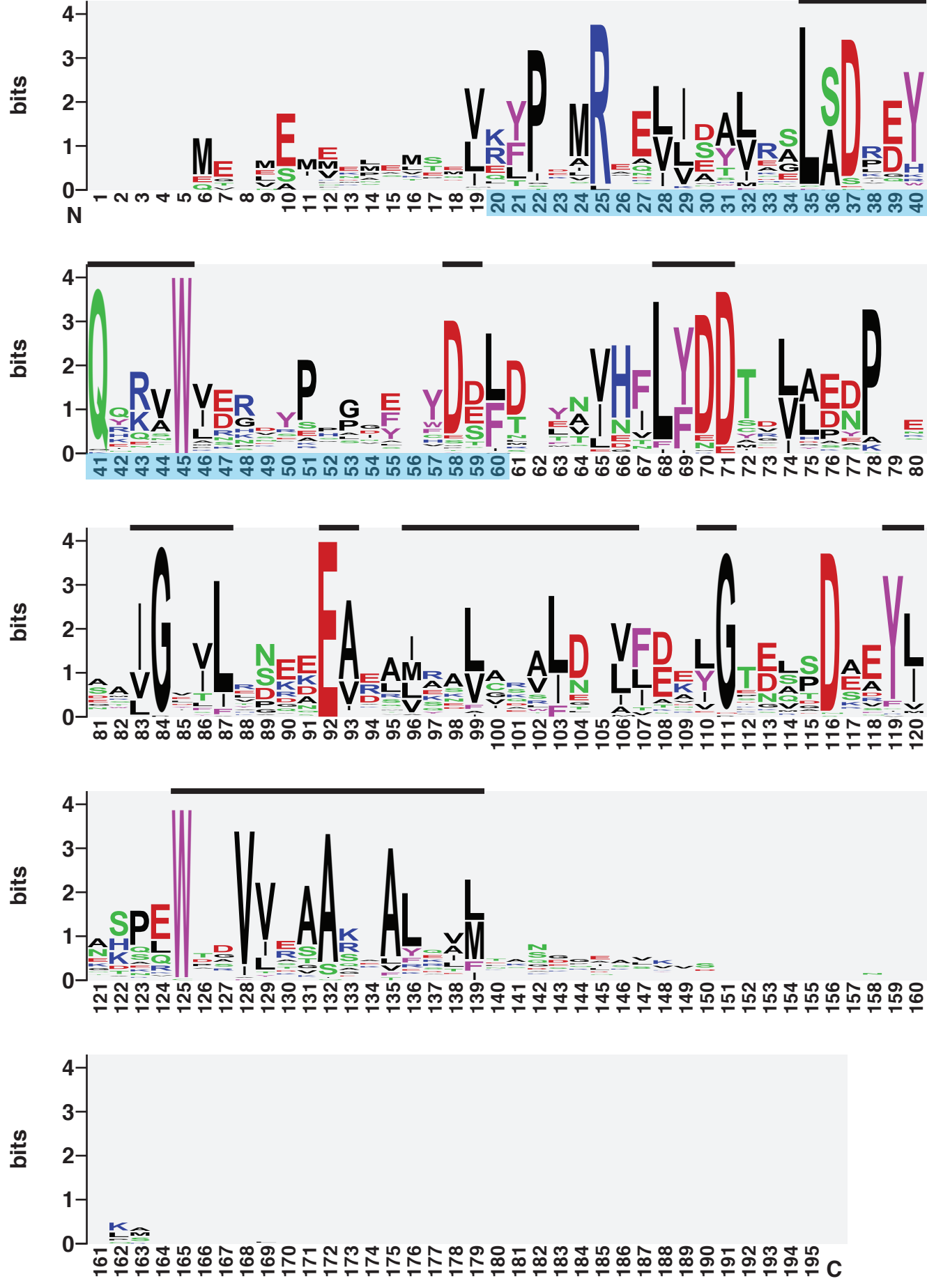

Fig. S2

Supplement: Supplementary Figure 2 — Sequence analysis of 380 predicted CRCAs. Sequence logos (Crooks et al., 2004) depict complete protein alignment generated using MUSCLE, default parameters (Edgar, 2004). Information for all proteins is provided in Table S2 . Regions shown in Figures 3 are denoted with a bar above the logos. Amino acid coloring as follows: black, hydrophobic; red, negatively charged; green, hydrophilic; purple, aromatic; blue, positively charged. Sequence spanning the adjusted start site of REIS_1423 (Rickettsia buchneri) insertion is shown in blue. [file DataSheet_3.pdf]

**rCRCT/CRCA-1 is found in a region of recombination adjacent to the SecA gene**

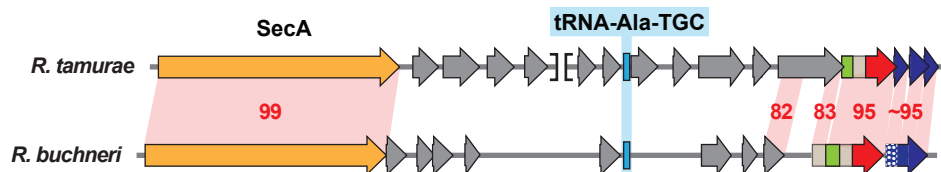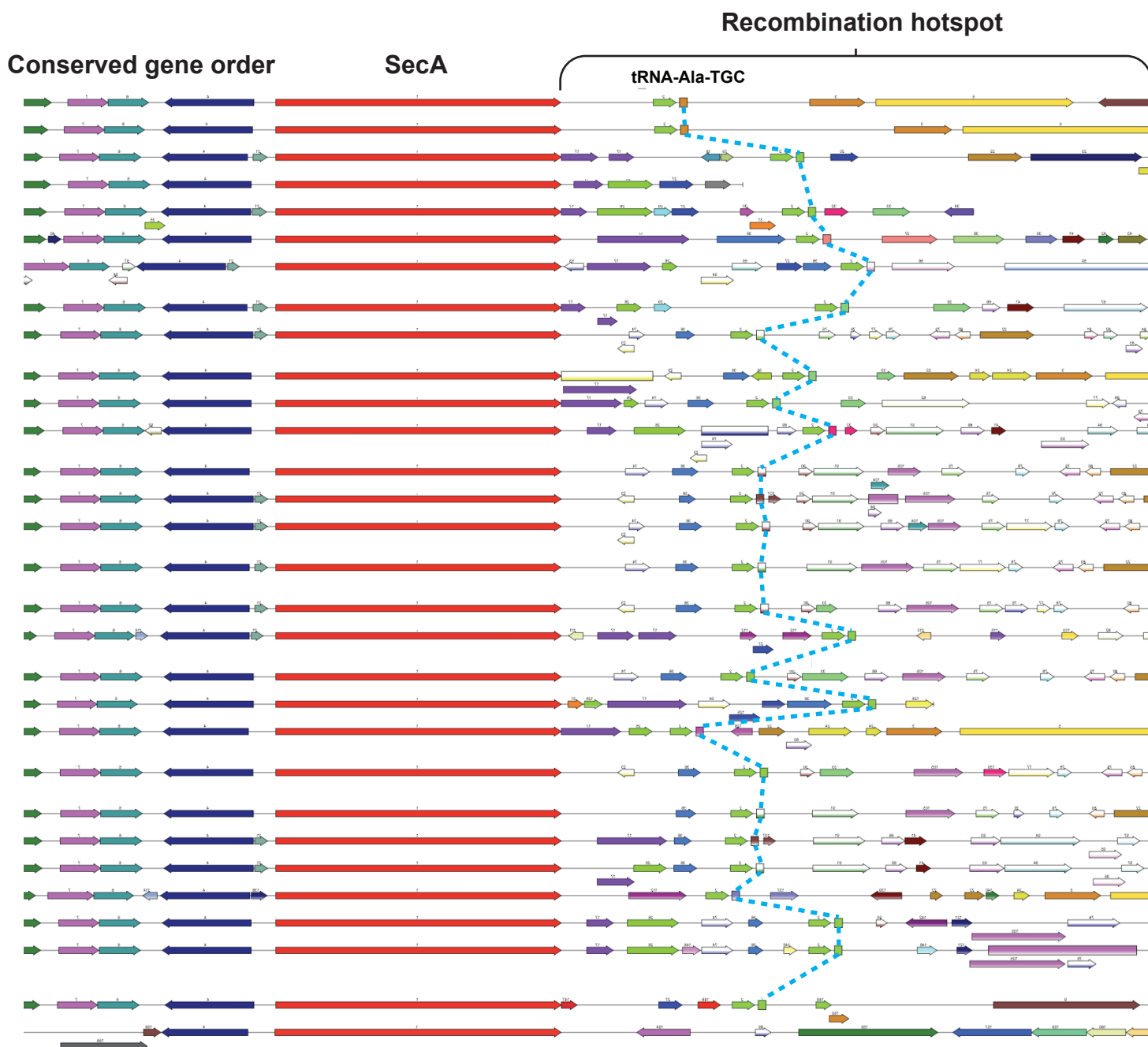

Supplement: Supplementary Figure 3 — Rickettsia CRCT/CRCA modules occur in three recombination hotspots. (A) rCRCT/CRCA-1 occurs in a recombination hotspot near the SecA and tRNA-AlaTGC genes. (B) rCRCT/CRCA-2 occurs in a recombination hotspot near the BamA and tRNA-ThrCGT genes. A second TA module, rCRCT/CRCA-3a, also occurs in this region and is distinct from rCRCT/CRCA-1 and rCRCT/CRCA-2 (cd20695: CdiA-CT_5T87E_Ct, cd20694: CdiI_Ct-like). (C) rCRCT/CRCA-3b proteins are analogous to rCRCT/CRCA-3a and occur in a subset of Rickettsia genomes between cyoB and cyoA, which encode the cytochrome c oxidase subunits I and II, respectively. [file DataSheet_4.pdf]

F

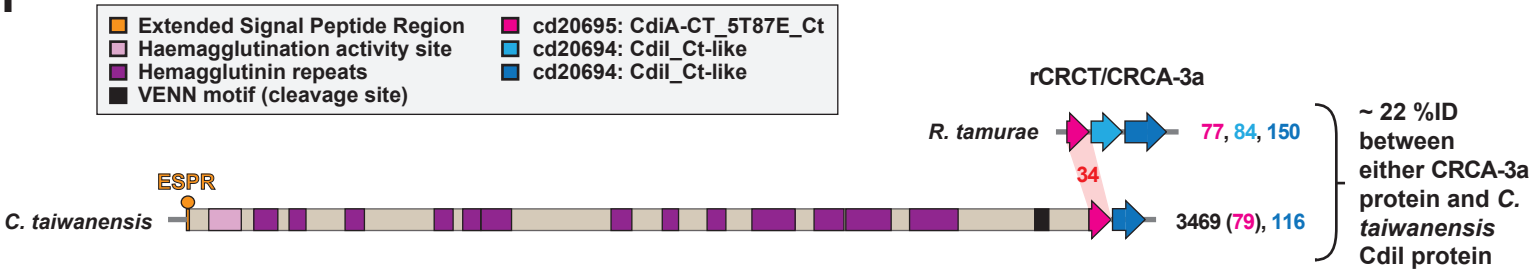

G

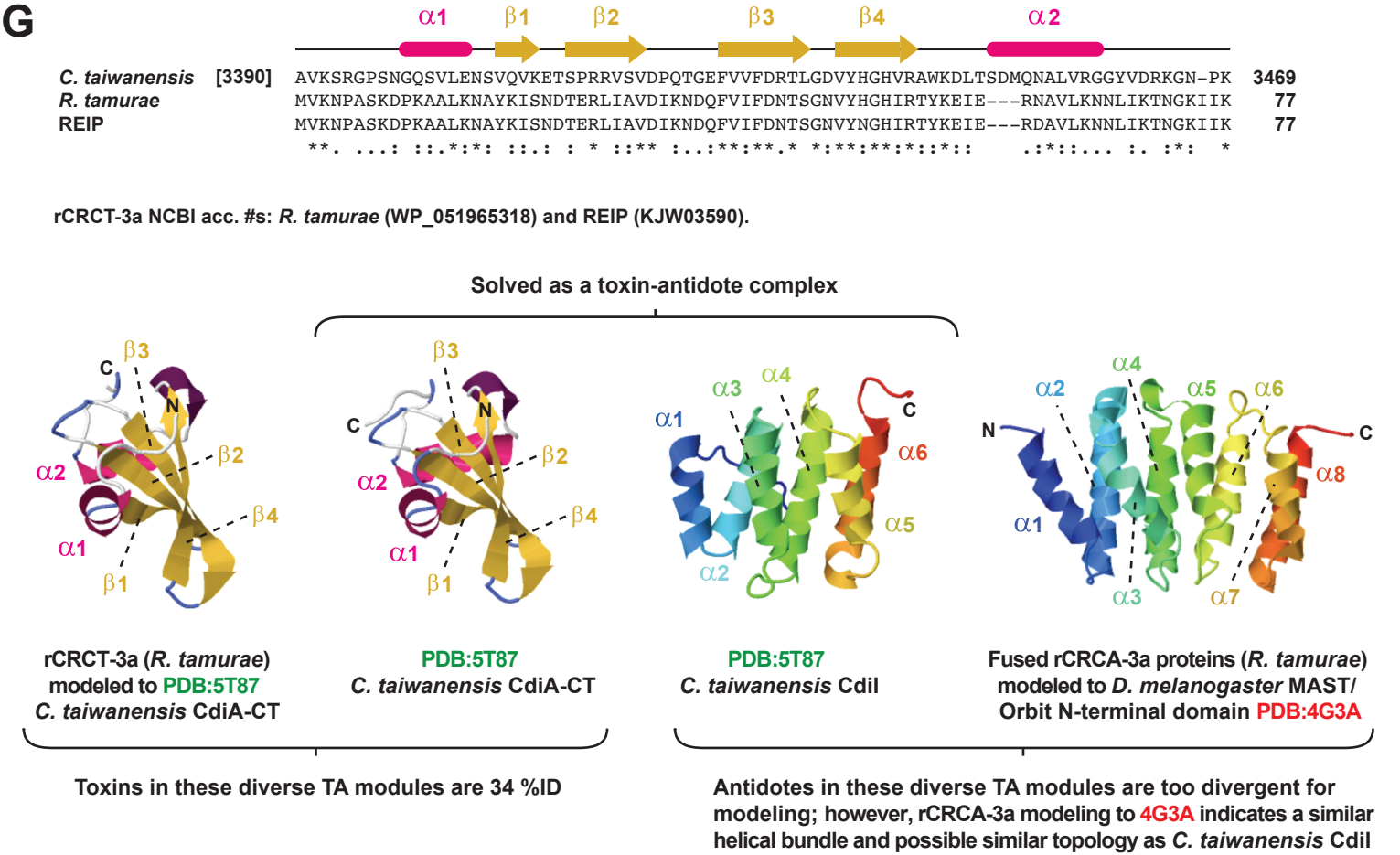

Fig. S4

Supplement: Supplementary file 9 [file DataSheet_9.pdf]

Fig. S5

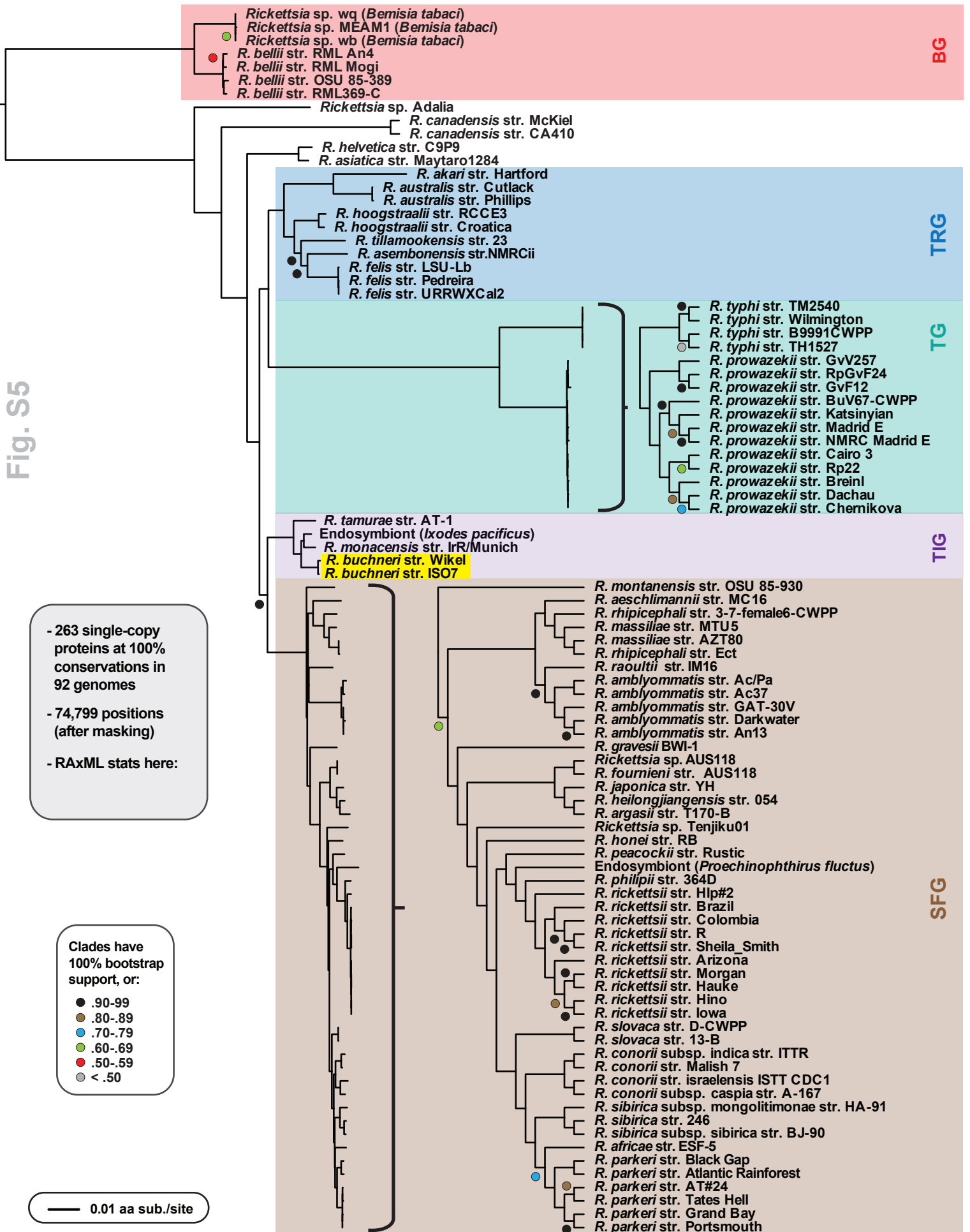

Supplement: Supplementary Figure 5 — Rickettsia genome-based phylogeny estimation. Rickettsia groups follow previous classification (Gillespie et al., 2007), except that we recognize Tamurae/Ixodes Group (TIG) rickettsiae as a distinct clade from SFG rickettsiae. R. buchneri is highlighted. Phylogeny was estimated for 92 Rickettsia genomes; gray inset described details (see “Materials and Methods” for more details). Branch support was assessed with 1,000 pseudoreplications. [file DataSheet_10.pdf]
