# Supplementary material for: Cryptic Genes for Interbacterial Antagonism Distinguish Rickettsia Species Infecting Blacklegged Ticks From Other Rickettsia Pathogens
Source: Front Cell Infect Microbiol. 2022 May 3;12:880813. doi: 10.3389/fcimb.2022.880813 (PMC9111745; doi:10.3389/fcimb.2022.880813)

Fig. S3

B

rCRCT/CRCA-2 is found in a region of recombination adjacent to the BamA gene

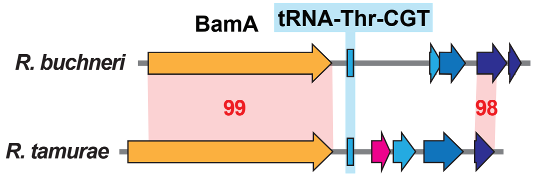

rCRCT/CRCA-3a is also found in this region, indicating multiple insertion of distinct CRCT/CRCAs

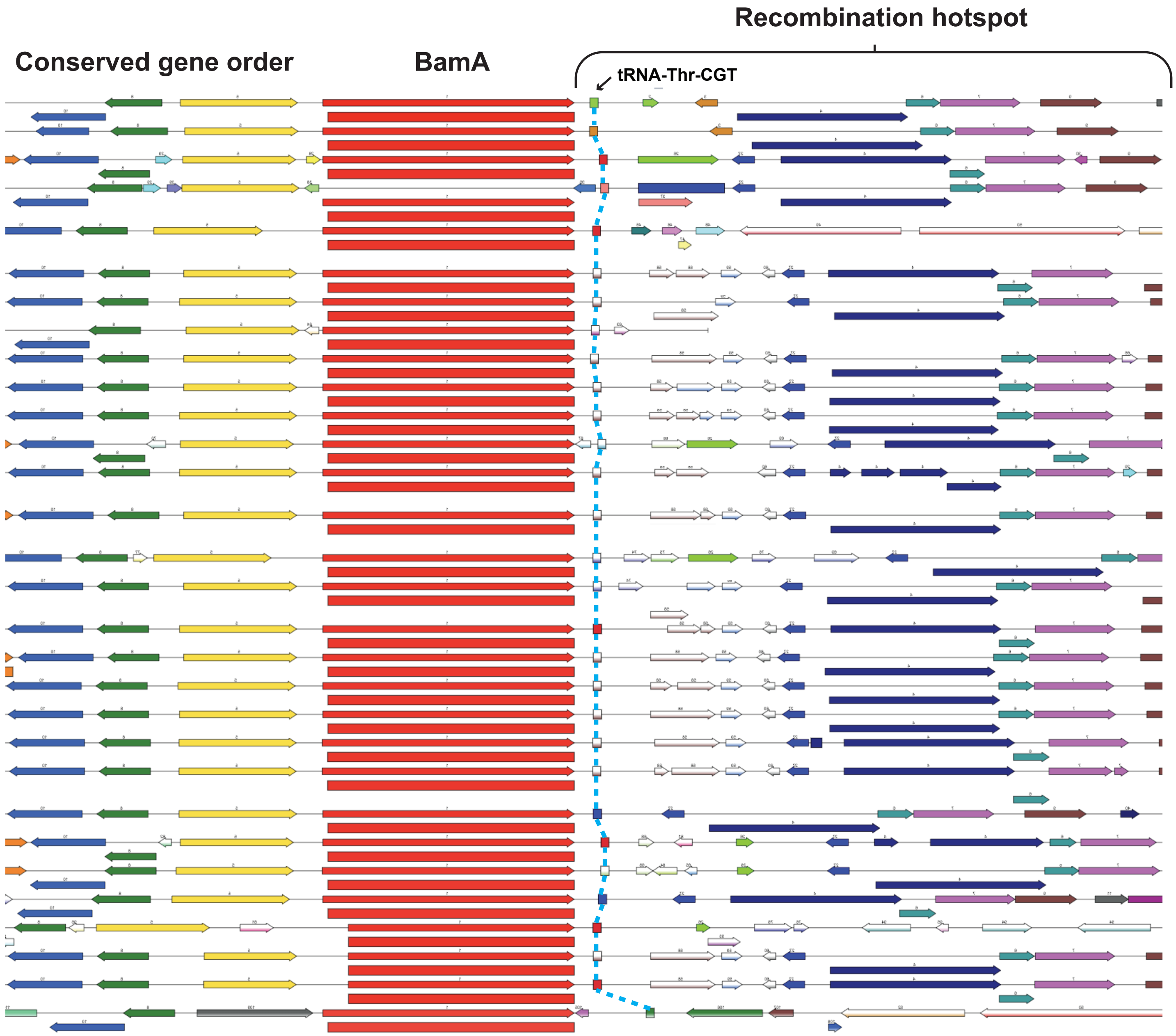

Supplement: Supplementary file 5 [file DataSheet_5.pdf]
