# Supplementary material for: Cryptic Genes for Interbacterial Antagonism Distinguish Rickettsia Species Infecting Blacklegged Ticks From Other Rickettsia Pathogens
Source: Front Cell Infect Microbiol. 2022 May 3;12:880813. doi: 10.3389/fcimb.2022.880813 (PMC9111745; doi:10.3389/fcimb.2022.880813)

Fig. S3

C

rCRCT/CRCA-3a is similar to rCRCT/ CRCA-3b between subunits I and II of cytochrome *c* oxidase

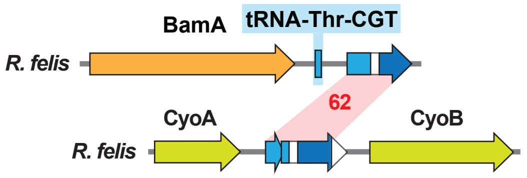

No toxins occur with CRCA-3b and this region has little recombination relative to SecA and BamA

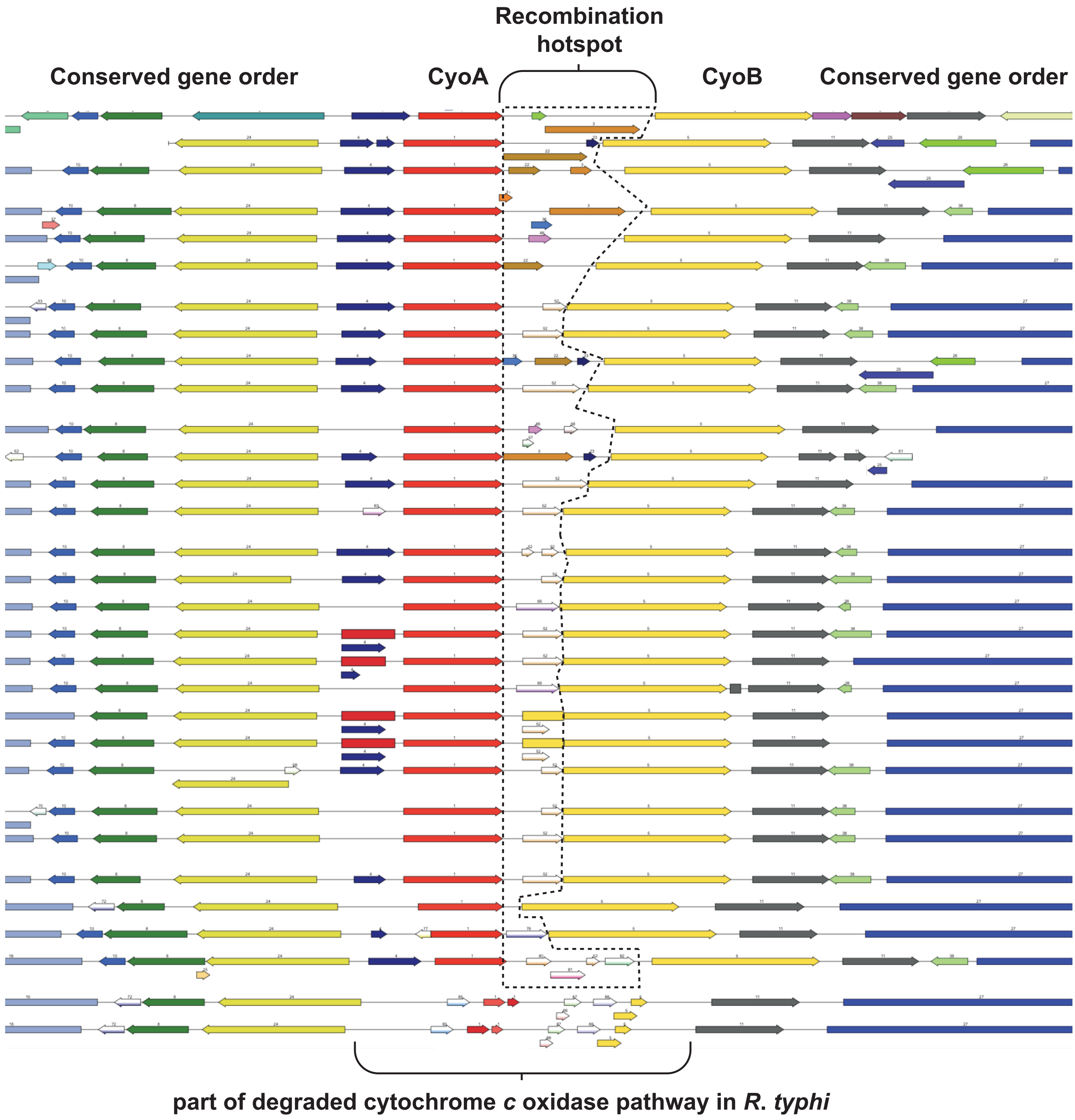

Supplement: Supplementary file 6 [file DataSheet_6.pdf]
