# Supplementary material for: Cryptic Genes for Interbacterial Antagonism Distinguish Rickettsia Species Infecting Blacklegged Ticks From Other Rickettsia Pathogens
Source: Front Cell Infect Microbiol. 2022 May 3;12:880813. doi: 10.3389/fcimb.2022.880813 (PMC9111745; doi:10.3389/fcimb.2022.880813)
Supplement: Supplementary Figure 4 — Evidence for recurrent integration of diverse CRCT/CRCA modules in Rickettsia genomes at recombination hotspots. (A) Subjects (n = 40) retrieved from a Blastp search against the NCBI nr database using five concatenated R. buchneri proteins (REIS_1427-REIS_1423) as the query. Bottom, a portion of the large protein (PFX12133, 2285 aa) from the coral Stylophora pistillata shares similarity with these smaller Rickettsia proteins. (B) rCRCT-2 proteins are analogous to rCRCT-1 proteins. Alignment performed using MUSCLE with default settings (Edgar, 2004). (C) rCRCA-3 proteins recovered from Blastp searches using R. bellii rCRCA-3b as a query (the largest rCRCA-3 protein). na, sequences not recovered in Blastp searches against the NCBI ‘Rickettsia’ database but retrieved from PATRIC (asterisks denote PATRIC Local Family IDs). Subjects are listed accordingly to their placement in the phylogeny presented in Figures 5E . Colored boxes unite rCRCA-3 proteins recombined within the same genome. (D) rCRCT-3a HaloBalst analysis. R. tamurae rCRCT-3a (WP_051965318) was used as the query. Concentric halos depict hierarchical taxonomic databases increasing in divergence from the center. Average Sm score (see text for details) for all subjects and top ten subjects are provided, with highest score per database highlighted. ‘na’, not applicable. (E) Top 25 subjects from the Blastp search against ‘Proteobacteria’ using R. tamurae rCRCT-3a as the query. (F) Comparison of the Cupriavidus taiwanensis str. DSM 17343 CdiA toxin (Uniprot acc. B3R1C1) to rCRCT-3a of R. tamurae. Domains were predicted with SMART (Letunic and Bork, 2017). Amino acid similarity (%ID, red shading) was assessed using Blastp. (G) Structural analysis of a rCRCT/CRCA-3a module. (top) Alignment of residues 3391-3469 of C. taiwanensis CdiA, R. tamurae rCRCT-3a, and REIP rCRCT-3a. Structural information from C. taiwanensis CdiA (PDB:5T87) (Kryshtafovych et al., 2018) is provided at top. Alignment performed using MUSCLE wit [file DataSheet_7.pdf]

A

	Max Score	Max Score	Query Cover	E value	Per. ident	Acc. Len	Accession
Hypothetical protein	*Rickettsia tamurae*	169	32%	6.00E-50	100	90	WP_152540701.1
	Rickettsia japonica	155	30%	2.00E-43	97.4	181	WP_014120923.1
	Rickettsia conorii	154	30%	9.00E-43	96.1	181	WP_014014523.1
	Rickettsia argasii	153	30%	1.00E-42	96.1	181	WP_045806003.1
	Rickettsia fournieri	152	30%	4.00E-42	96.1	181	WP_103896886.1
	Rickettsia slovaca	150	30%	6.00E-42	94.81	144	WP_014419876.1
	Rickettsia gravesii	151	30%	1.00E-41	94.81	181	WP_017442373.1
	Rickettsia peacockii	148	30%	6.00E-41	93.51	144	WP_085065668.1
	Rickettsia sp. Tenjiku01	149	30%	9.00E-41	93.51	181	WP_064429221.1
	Rickettsia sibirica	146	30%	2.00E-40	92.21	144	WP_004998075.1
	Rickettsia sibirica	146	30%	3.00E-40	92.21	144	WP_016770294.1
	Rickettsia philipii	145	30%	4.00E-40	92.21	144	WP_014364856.1
	Rickettsia tamurae	137	29%	1.00E-35	92	248	WP_215426163.1
	Rickettsia conorii subsp. raoultii	100	20%	3.00E-23	94.12	81	AJQ51990.1
	Rickettsia conorii	99.8	20%	9.00E-23	92.16	92	WP_014014522.1
	Rickettsia argasii	98.6	20%	2.00E-22	92.16	92	WP_045806002.1
	Rickettsia honei	95.9	20%	2.00E-21	90.2	86	WP_032850298.1
	Rickettsia sibirica	95.9	20%	2.00E-21	90.2	92	WP_004998077.1
	Rickettsia sibirica	95.9	20%	2.00E-21	90.2	92	WP_026054999.1
	Rickettsia slovaca	95.9	20%	3.00E-21	90.2	92	WP_014419875.1
	Rickettsia parkeri	97.4	37%	4.00E-21	92.16	158	QWB86932.1
	Rickettsia parkeri	97.4	38%	4.00E-21	92.16	157	WP_012719858.1
	Rickettsia parkeri	96.3	37%	1.00E-20	90.2	158	WP_146709252.1
	Rickettsia conorii	92.8	20%	3.00E-20	88.24	92	WP_010977480.1
	Rickettsia peacockii	92.8	20%	4.00E-20	88.24	92	WP_085065667.1
	Endosymbiont of *Proechinophthirus fluctus*	91.7	38%	1.00E-19	86.27	90	KYP98281.1
	Rickettsia japonica YH	73.6	15%	5.00E-13	87.18	82	BAK96855.1
	Rickettsia conorii	72.4	16%	2.00E-12	85.37	82	WP_029374682.1
*	*Stylophora pistillata*	77.8	46%	4.00E-12	42.62	2285	PFX12133.1
Hypothetical protein	*Rickettsia* endosymbiont of *Ixodes pacificus*	70.1	13%	6.00E-12	100	47	KJW02813.1
	Rickettsia monacensis	65.9	13%	2.00E-10	96.97	33	CEO17499.1
	Rickettsia conorii	65.5	22%	5.00E-10	64.29	67	WP_014014521.1
	Rickettsia felis	65.1	13%	7.00E-10	85.29	69	WP_039594829.1
	Rickettsia fournieri	62.8	13%	3.00E-09	87.88	52	WP_198913008.1
	Rickettsia japonica YH	60.5	13%	2.00E-08	84.85	44	BAK96854.1
	Rickettsia argasii	60.5	13%	2.00E-08	84.85	52	WP_186786312.1
	Rickettsia conorii	59.7	13%	5.00E-08	84.85	52	WP_156860613.1
	Rickettsia philipii str. 364D	58.5	13%	9.00E-08	87.88	33	AFB26505.1
	Rickettsia argasii T170-B	54.3	11%	4.00E-06	83.33	41	KJW03890.1

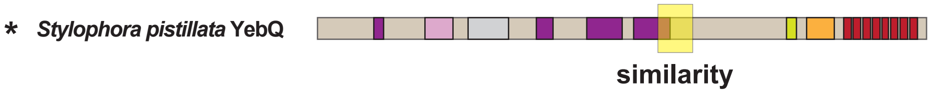

B

```
rCRCA-1  C R. buchneri    -----------LTIQCLE-EIKDAFEELADFEKQQKTWPKNSEKHPHSYWDSLMFYVLENLYNDCE--------------  54
         S R. tamurae     MDYSQQTLKFKINYPMFRMEIKDAFEELADFEKQQKTWPKNSEKHPHS-----MFYVLENLYNDCE--------------  61
rCRCA-2  F R. tamurae     ---------MWLLSPALRWEIFATFKALANKELQLTKWVDS--KYKHSFWDELCF-CIDTLFNDANA-------------  55
         S R. buchneri    --------MMWLLSPALRWEIFATFKELANKELQLTKWVDS--KYKHSFWNELCF-CIDTLFNDANA-------------  56
         C REIP           ----MKHRMMWLLSPALRWEIFATFKELANKELQLTKWVDS--KYKHSFWDELCF-CIDTLFNDANA-------------  60
         S R. monacensis  --------MMWLLSPALRWEIFATFKELANKELQLTKWVDS--KYKHSFWDELCF-CIDRLFNDVRQNITNMNYAFVLIG  69
                                     :    :  **  :*: **: * *  ..* ..  *: **      *  :: *:**

rCRCA-1  C R. buchneri    ----LHEYKPENIGELFYNEEEAKQVYEFCKWFNKLTENIGENQPDAAYLNHPE-------------WHRVYSGAKKLFE  117
         S R. tamurae     ----LHEYKPENIGELFYNEEEAKQVYEFCKWFNKLTENIGENQPDAAYLNHPECIGFIAVQKSFLNMHRVYSGAKKLFE  137
rCRCA-2  F R. tamurae     ----LDKTSQIKIGLIMYIKKK                                                            73
         S R. buchneri    ----LDKTSQIKIGLTMYNQEEVEKVNKFSHFFYDLTEEIEGEKPDYRILRSPT-------------VAKDYR-------  112
         C REIP           ----LDKTSQIKIGLTMYNQEEVEKVNKFSHFFYDLTEEIEGEKPDIEYYGHPQ-------------WQKIIDDATELVE  123
         S R. monacensis  FLMMLDKTSQIKIGLTMYNQEEVEKVNKFSHFFYDLTEEIEGEKPDIEYYGHPQ-------------WQKIIDDATELVE  136
                              * :  .   :**  :* :::.::* :*.::* .***:*  ::**      *                 .

rCRCA-1  C R. buchneri    LMDKNKNIILVKSMQF---IIKHNRKKLME-------------KNNNLMF  151
         S R. tamurae     LMDKND-----KKYNFSEEYAAYNKAQWEE---------------TYGKK  167
rCRCA-2  F R. tamurae                                                        73
         S R. buchneri    -MKQNN-----KANDFSECLAQFNQFNIDGYEDYDDFAIAENTKNGYKLR  156
         C REIP           IMKQNN-----KANDFSECLAQFNQFNIDGYEDYDDFAIAENTKNGYKLR  168
         S R. monacensis  IMKQNN-----KANEFSECLAQFNQFNIDEYEDYDDFAIAENTKNGYKLR  181
                           *.:*.      *   :*
```

C = Complete

S = Split (multiple CDS)

F = Fragment

M Start site of additional CDS

rCRCA-2 NCBI acc. #s: *R. tamurae* (WP_032139358); *R. buchneri* (EER22830, EER22831); REIP (KJW03593); *R. monacensis* (CEO17090, CEO17089).

Fig. S4
