# Supplementary material for: Cryptic Genes for Interbacterial Antagonism Distinguish Rickettsia Species Infecting Blacklegged Ticks From Other Rickettsia Pathogens
Source: Front Cell Infect Microbiol. 2022 May 3;12:880813. doi: 10.3389/fcimb.2022.880813 (PMC9111745; doi:10.3389/fcimb.2022.880813)
Supplement: Supplementary file 8 [file DataSheet_8.pdf]

C

Group	Taxon	Max Score	Query Cover	E value	%ID	Acc. Len	Accession
rCRCA-3a	*Rickettsia helvetica C9P9*	na	na	na	na	99	780_00002939 *
	Rickettsia helvetica C9P9	na	na	na	na	154	780_00001997 *
	Rickettsia asiatica	77.4	19%	3.00E-14	65.52	68	BBJ31155.1
	Rickettsia akari	55.1	15%	4.00E-06	52.17	50	WP_012013424.1
	Rickettsia akari	95.5	18%	5.00E-21	83.33	60	WP_012149527.1
	Rickettsia australis str. Cutlack	52	15%	3.00E-05	47.83	46	AFC71639.1
	Rickettsia australis str. Cutlack	57.4	14%	4.00E-07	69.05	51	AFC71642.1
	Rickettsia hoogstraalii str. RCCE3	173	54%	1.00E-49	58.28	179	KJV81432.1
	Rickettsia asembonensis	248	79%	2.00E-78	56.96	236	WP_041078980.1
	Rickettsia felis URRWXCal2	272	82%	9.00E-88	58.63	245	AAY61973.1
	Rickettsia tamurae	154	45%	1.00E-42	60.74	150	WP_032139359.1
	Rickettsia tamurae	101	28%	6.00E-23	58.33	84	WP_032139360.1
	Rickettsia endosymbiont of Ixodes pacificus	111	29%	2.00E-26	65.17	113	WP_197069219.1
	Rickettsia endosymbiont of Ixodes pacificus	87.8	24%	4.00E-18	58.9	75	WP_045804188.1
	Rickettsia monacensis IrR/Munich	na	na	na	na	42	780_00003822 *
	Rickettsia monacensis IrR/Munich	82.4	18%	4.00E-16	70.91	59	CDI29282.1
	Rickettsia monacensis	80.9	30%	3.00E-15	48.89	89	WP_023507580.1
	Rickettsia buchneri	112	30%	3.00E-27	64.84	99	WP_008581282.1
	Rickettsia buchneri	79.3	28%	2.00E-14	50	99	WP_008581283.1
	Rickettsia bellii	602	100%	0	100	300	WP_011477556.1
rCRCA-3b	*Rickettsia hoogstraalii str. RCCE3*	330	83%	4.00E-110	68.25	263	KJV80994.1
	Rickettsia asembonensis	109	40%	1.00E-25	52.89	128	KIJ89243.1
	Rickettsia felis URRWXCal2	322	80%	2.00E-107	70.95	239	AAY61479.1
	Rickettsia tamurae	na	na	na	na	267	780_00002380 *
	Rickettsia endosymbiont of Ixodes pacificus	186	58%	1.00E-54	55.56	175	WP_197069193.1
	Rickettsia monacensis IrR/Munich	44.7	10%	0.012	71.88	39	CDI29539.1
	Rickettsia monacensis IrR/Munich	241	73%	7.00E-76	55.25	221	CDI29540.1
	Rickettsia buchneri	67.4	18%	1.00E-10	48.21	59	KDO03272.1
	Rickettsia buchneri	139	38%	3.00E-37	59.48	117	KDO03273.1
	Rickettsia buchneri	66.2	17%	6.00E-10	66.04	74	KDO03274.1

D

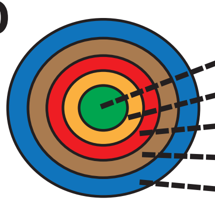

Taxon database		Subj.	x̄ *Sm*	top 10 *Sm*
Rickettsiales,	"Rick"	1	na	na
Alphaproteobacteria (- Rickettsiales),	"Alph"	1	na	na
Proteobacteria (- Bacteria),	"Prot"	154	1625.03	2348.56
Bacteria (- *Proteobacteria*),	"Bact"	60	1622.45	2203.55
- Bacteria,	"- Bact"	0	na	na

Unlike rCRCT-1, rCRCT-3a is more similar to toxins from *Proteobacteria* than other bacteria

Fig. S4

E

Annotation [taxon]	Max Score	Query Cover	E value	%ID	Acc. Len	Accession
Exoprotein, adhesin or hemolysin [*M. bovoculi*]	62.4	96%	7.00E-11	40.26	103	AKG19840.1
Hypothetical protein [*M. catarrhalis*]	61.2	96%	2.00E-10	41.56	103	WP_003659587.1
Adhesin [*M. catarrhalis*]	61.2	96%	2.00E-10	41.56	101	WP_182997174.1
Hypothetical protein [*M. catarrhalis*]	60.8	97%	2.00E-10	38.46	97	WP_064602974.1
Hypothetical protein [*M. catarrhalis*]	60.8	97%	2.00E-10	38.46	103	WP_064643002.1
Adhesin [*M. catarrhalis*]	60.8	97%	2.00E-10	38.46	103	WP_120655496.1
Adhesin [*M. catarrhalis*]	60.8	97%	2.00E-10	38.46	101	WP_196758664.1
Hypothetical protein [*M. bovoculi*]	62	96%	3.00E-10	40.26	181	WP_080947368.1
Hypothetical protein [*M. catarrhalis*]	59.3	97%	8.00E-10	37.18	103	WP_064622593.1
Hypothetical protein [*M. catarrhalis*]	59.3	97%	1.00E-09	37.18	103	WP_064607916.1
Adhesin [*M. catarrhalis*]	58.9	96%	1.00E-09	40.26	103	WP_120655766.1
Hypothetical protein [*M. catarrhalis*]	61.2	96%	1.00E-09	41.56	230	MPY09055.1
Adhesin [*M. catarrhalis*]	58.5	88%	2.00E-09	44.12	104	RKM14712.1
Putative hemagglutinin protein [*M. catarrhalis*]	59.3	97%	2.00E-09	38.46	144	OAV35577.1
Adhesin [*M. catarrhalis*]	58.2	88%	2.00E-09	44.12	97	WP_183001834.1
S-type pyocin domain protein [*P. fluorescens*]	61.6	97%	2.00E-09	34.62	413	WP_150710635.1
Adhesin [*M. catarrhalis*]	57.4	97%	4.00E-09	35.9	101	WP_204281388.1
Hypothetical protein [*M. catarrhalis*]	59.7	97%	5.00E-09	38.46	218	WP_081258233.1
S-type pyocin domain protein [sp. Ant30-3]	60.5	98%	6.00E-09	35.53	412	WP_028622128.1
S-type pyocin domain protein [*P. frederiksbergensis*]	59.3	97%	1.00E-08	37.5	415	WP_076031368.1
Hypothetical protein [*P. farris*]	55.5	79%	2.00E-08	39.34	76	WP_225922902.1
Adhesin [Pseudomonas]	56.2	92%	2.00E-08	33.8	103	WP_102819864.1
S-type pyocin domain protein [sp. GL-B-26]	58.9	97%	2.00E-08	37.5	412	WP_223430912.1
S-type pyocin domain protein [*P. fluorescens*]	58.9	97%	2.00E-08	37.5	412	WP_046048933.1
★ Crystal structure CDI complex [*C. taiwanensis*]	55.1	81%	2.00E-08	31.82	76	5T87_E

■ ***Moraxella*** ■ ***Pseudomonas*** ■ ***Cupriavidus*** ★ **Further comparisons shown in Fig. S4F, G**
