# Supplementary material for: Cryptic Genes for Interbacterial Antagonism Distinguish Rickettsia Species Infecting Blacklegged Ticks From Other Rickettsia Pathogens
Source: Front Cell Infect Microbiol. 2022 May 3;12:880813. doi: 10.3389/fcimb.2022.880813 (PMC9111745; doi:10.3389/fcimb.2022.880813)
Supplement: Supplementary Figure 6 — REIS_1424 contains a ubiquitin-associated domain demarcated from the C-terminal toxin by two predicted VENN motifs. (Mueller and Feigon, 2002); (E) Schemas for a few. Accession nos. Described domains here or in the M&M and get refs from SMART (Letunic and Bork, 2017). Wolbachia supergroups are within colored ellipses. Ctenocephalides felis- [file DataSheet_11.pdf]

Fig. S6

>EER22217.1 hypothetical protein REIS_1424 [Rickettsia endosymbiont of Ixodes scapularis]

```
MEFISSALKPQQASAFSAGEEELTAKQQEELKRQEEERLQEILDEPGVEPVYPEMLVLSLISAPFRLVKS
LYGLFKDQIIDLVSDSYKDKAIKLSREIDNNTIDVSNNITKLQKHIEDITGVKLDNLELEKYGKYLEEYE
IWVQLPEIVKKIPEKLGFPKFSEKGTGLLYGDKVNGMRIMKGRSNVEFSHQKVDYAVIVDKGCRIDKYGN
QLRYNSNTKVTEVIDPVSNKIIKEIPGFKLKKIPEAHINLEEWIKKWITHNKP
```

- Ubiquitin associated domain
- VENN motif (manual prediction)
- CRCT (CDI-like/Rhs-like CT toxin)

Rickettsia buchneri REIS_1424 N-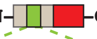-C 263

REIS_1424	EEERLQEILD E.PGVEPVYP EMLVLSLISA PFR.LVKSLY GLFK	CdiA-like/Rhs-like C-terminal toxin (CRCT)
YBE7_YEAST_1	KSLAVEELSG M..GFTEE.. .EAHNALEKC NWD.LEAATN FLLD	EH domain-containing and endocytosis protein 1
HSU75362-1	DESSVMQLAE M..GFPLE.. .ACRKAVYFT GNMGAEVAFN WIIV	Solution structure of tandem UBA of USP13
UBPC_YEAST_1	NQCSISQLIE M..GFTQN.. .ASVRALFNT GNQDAESAMN WLFQ	Ubiquitin carboxyl-terminal hydrolase 12
UPBA_DICDI	NSQDVDNIIG M..GFTDS.. .QAKLALKNT KGN.LERAAD WLFS	Ubiquitin carboxyl-terminal hydrolase A
RNMARK1_1	DAKRIDIMVT M..GFARD.. .EINDALVSQ KYD.EVMATY ILLG	Serine/threonine-protein kinase MARK1 isoform X1
UBPC-YEAST_2	DEVSLTSMLS M..GLNPN.. .LCRKALILN NGD.VNRSVE WVFN	Ubiquitin carboxyl-terminal hydrolase 12
DMUBCD4_1_1	CDSKIQRLRD M..GIDEH.. .EARAVLSKE NWN.LEKATE GLFS	AT12021p
HSDKG24_1	FEEKVKQLID I.TGKNQD.. .ECVIALHDC NGD.VNRAIN VLLE	Ubiquitin-associated protein 2-like
CET12G3_4_1	TEKTFDRLLE M..GFSYN.. .VTVAAIRAN GSN.LEMCLQ ALLH	SeQueSTosome related
KP78_HUMAN_1	DQKRIDIMVG M..GYSQE.. .EIQESLSKM KYD.-EITAT YLLL	MAP/microtubule affinity-regulating kinase 3
OSU63530-1	EDEAILRLEP M..GFDRA.. .LVLDVFFAC NKD.EQLAAN YLLD	osRAD23
CBL_MOUSE_1	LSSEIERLMS Q..GYSYQ.. .DIQKALVIA HNN.IEMAKN ILRE	E3 ubiquitin-protein ligase CBL
MMU27462+1_3	SQESINQLVY M..GFDTV.. .VAEAALRVF GGN.VQLAAQ TLAH	Solution structure of RSGI RUH-012
UBC1_BOVIN_1	YTKKIENLCA M..GFDRN.. .AVIVALSSK SWD.VETATE LLLS	Ubiquitin-conjugating enzyme E2 K
MMU27462_1_1	DPSKVHNLLQ L..GFTAQ.. .EARLGLRAC DGN.VDHAAT HISN	Nub1 protein
RA23_YEAST_1	RNETIERIME M..GYQRE.. .EVERALRAA FNN.PDRAVE YLLM	UV excision repair protein RAD23
HSNADGLCT_1	PATDIDRILR A..GFTLQ.. .EALGALHRV GGN.ADLALL VLLA	Regulatory solute carrier protein family 1 member 1
HS467511_1	LIESLSQMLS M..GFSDEG. GWLTRLLQTK NYD.IGAALD TIQY	NMR structure of the UBA domain of p62 (SQSTM1)
HUMHHR23A_1_1	YETMLTEIMS M..GYERE.. .RVVAALRAS YNN.PHRAVE YLLT	Solution structure (Internal UBA Domain of HHR23A)
ATHAKIN10A_1_1	DEEILQEVIN M..GFDRN.. .HLIESLRNR TQN.DGTVTY YLIL	Serine/threonine protein kinases, catalytic domain
HSSTPKEMK_1	DPRRTELMVS M..GYTRE.. .EIQDSLVGQ RYN.EVMATY LLLG	Serine/threonine-protein kinase MARK2 isoform X5
CEUT27A3_6	VRFVAGELML M..GFCEN.. .ACYRAAYYS NGN.VEIASN WLME	Ubiquitin specific protease 5 (Zn finger protein)
STSNF1PKN_1	DEEILQQVSR M..GLDRD.. .QLLDSLQKR IQD.DATVAY YLLY	SNF1-related protein kinase
CEU22183_1	RIEKLIQIFQ L..GFNKA.. .AILESVEKE KFE.DIHATY LLLG	H39E23, serine/threonine Kinase
ATTS1169_1_1	LEEKVKRLVE M..GFGDA.. .QVRSAIESS GGD.ENLALE KLCS	Ubiquitin-conjugating enzyme 27
HSU75362_1	PEEIVAIITS M..GFQRN.. .QAIQALRAT NNN.LERALD WIFS	Solution structure of tandem UBA of USP13
T24B8.7	YQDNQDMLIT M..GFKSE.. .EIDTALRLS NND.VEKAIQ YLTR	UBA domain-containing protein
YAUG_SCHPO	YAEQLSQLNE M..GFVDF.. ERNVQALRRS GGN.VQGAIE SLLS	Deubiquitination-protection protein dph1
YSCL9931_9_1	TRMIVERLTE I..GVSSD.. .EALLALQQN DMN.ENEAAG FLTR	UBA (putative ATP-dependent RNA helicase YLR419W)
CEF15C11_2	YASQLEQLQS M..GFSDR.. ARNVAALTAT FGD.LNAAVE RLLN	Ubiquilin
CEZK20_5	QEENVLAITG M..GYDRE.. .QTIAALRAA FWN.PDRAVE FLLN	UV excision repair protein RAD23
HS2671010_1	VDAKIAKLMG E..GYAFE.. .EVKRALEIA QNN.VEVARS ILRE	E3 ubiquitin-protein ligase CBL-B
SC130KBXV_53_1	DNQAVKSLLE M..GIPHE.. .VAVDALQRT GGN.LEAAVN FIFS	Solution structure of UBA of RUP1p
YEX3_YEAST_1	PEQTIKQLMD L..GFPRD.. .AVVKALKQT NGN.AEFAAS LLFQ	DNA damage-inducible protein 1
GTS1_YEAST	YSRQLAELKD M..GFGDT.. NKNLDALSSA HGN.INRAID YLEK	GTS1 transcription factor (heat resistance and flocculation)
CELC06E2_2_1	QKKKVDSLIE M..GFSRL.. .ESILALGGS DWN.LADAAE QLLE	Ubiquitin Conjugating enzyme
HUMORF_1_1	ELTALESLIE M..GFPRG.. .RAEKALALT GNQGIEAAMD WLME	UBA (UBX domain-containing protein 1 and similar proteins)
OSU63530_1	LEATIQSILE MGGGIWDR.. DIVLHALSAA FNN.PERAVE YLYS	osRAD23
MMU27462-1_2	RLENVNTLRG M..GYSTQ.. .AAKQALHQA RGN.LDDALK VLLS	Nub1 protein
CEF49C12_8	YEQQAATLRE F..GFEND.. ELIQLALEQA NGD.VQAAME FLID	UBA domain-containing protein
SPAC3F10_13_1	LDTKIKTLKN M..GVSES.. .DAKDSLERC GYD.VESAAE FIFS	UBA domain protein Ucp6
UPBA-DICDI	NQEVLDTLLS M..DFPLV.. .RCKKALLAT GGKDAELAMN WIFE	Deubiquitinating enzyme, peptidase C19 family
RF2P_DROME	INKSIHAMMA M..GFSNEG. AWLTQLLESV QGN.ISAALD VMNV	Ref(2) Pp protein
YD28_SCHPO	LNSKIAQLVS M..GFDPL.. .EAAQALDAA NGD.LDVAAS FLL-	UBA domain protein Mud1
HUMHHR23A-1_2	EKEAIERLKA L..GFPES.. .LVIQAYFAC EKN.ENLAAN FLLS	UV excision repair protein RAD23 homolog A
Consensus/60%	.ppplppLbs M..GFspp.. .psbpALp.s sss.hpbAhp bLbp	
	* * ** ** * *	

Saccharomyces cerevisiae; Homo sapiens; Dictyostelium discoideum; Rattus rattus; Drosophila melanogaster; Pseudonaja textilis; Caenorhabditis elegans; Oryza sativa Japonica Group; Mus musculus; Bos taurus; Arabidopsis thaliana; Piliocolobus tephrosceles; Solanum tuberosum; Schizosaccharomyces pombe
