# Supplementary material for: Cryptic Genes for Interbacterial Antagonism Distinguish Rickettsia Species Infecting Blacklegged Ticks From Other Rickettsia Pathogens
Source: Front Cell Infect Microbiol. 2022 May 3;12:880813. doi: 10.3389/fcimb.2022.880813 (PMC9111745; doi:10.3389/fcimb.2022.880813)
Supplement: Supplementary Figure 7 — Gene neighborhoods for CRCT/CRCA modules in diverse bacteria. (A-G) Genome neighborhoods for the CRCT/CRCA modules shown in and for (A) “Candidatus Jidaibacter acanthamoeba”, (B) Sphingobium chlorophenolicum, (C) Holosporales bacterium, (D) Pseudomonas orientalis, (E) Enterobacter sp. BIGb0383, (F) Streptomyces luteoverticillatus, (G) Sphaerisporangium krabiense. (H) Results of Blastp searches against these seven genomes (specific taxon databases at NCBI) using the CdiB protein of Escherichia coli (UniProtKB-Q3YL97). (I) Occurrence of CdiB and CdiB-like proteins in the major taxa in which CRCT/CRCA modules were identified. [file DataSheet_12.pdf]

Fig. S7

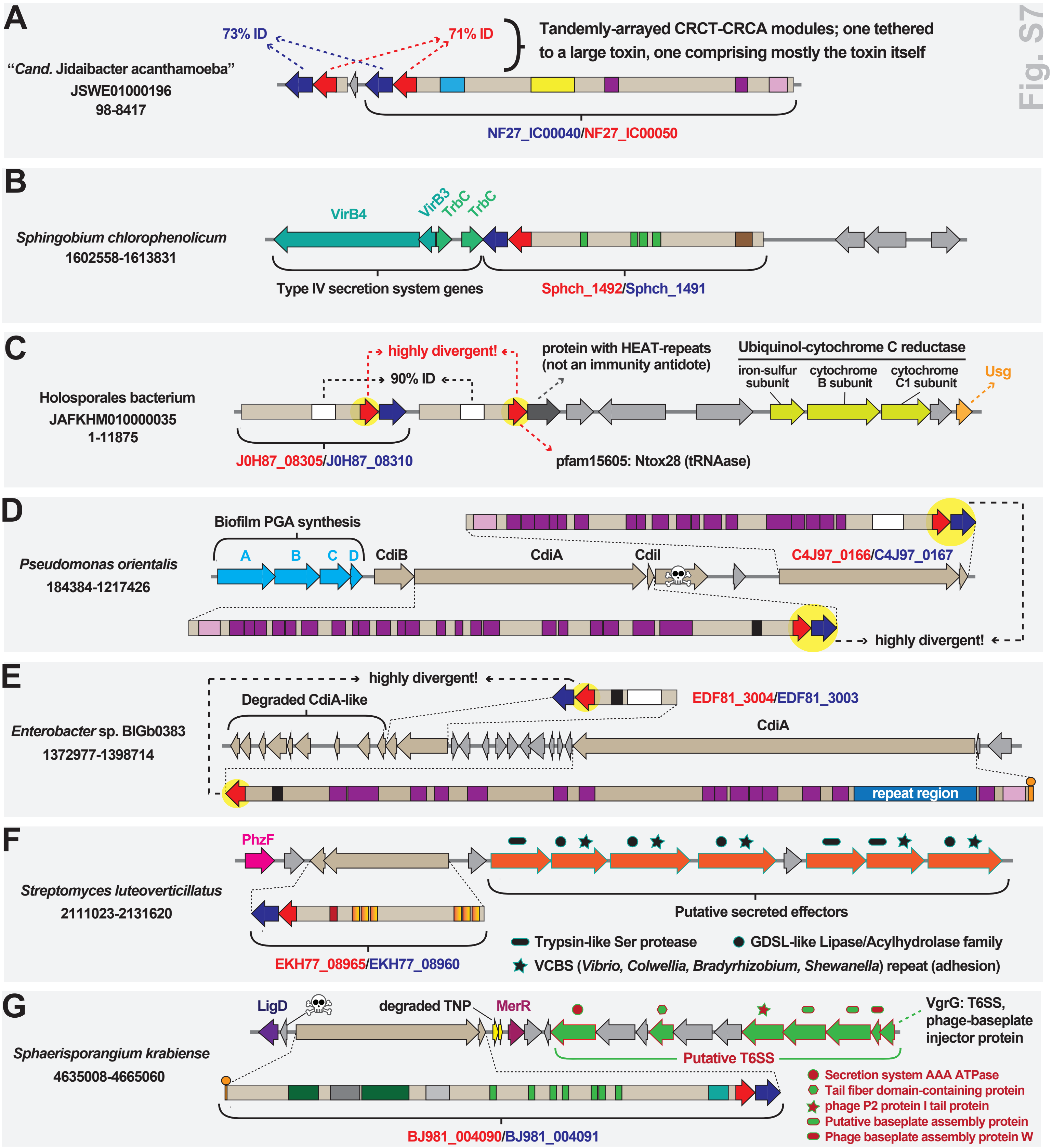

H

Taxon (genome)	Subjects (with > 80% query coverage)
"*Cand.* Jidaibacter acanthamoeba"	---
Sphingobium chlorophenolicum	---
Holosporales bacterium	---
Pseudomonas orientalis	C4J97_0161 (33 %ID)
Enterobacter sp. BIGb0383	EDF81_4644 (60 %ID); EDF81_3716 (29 %ID)
Streptomyces luteoverticillatus	---
Sphaerisporangium krabiense	---

I

Taxon	No.	No. > 80% query cov.
Rickettsiales	16	10 (no *Rickettsia* species)
Actinobacteria	12	2
Cyanobacteria	> 1000	> 1000 (widespread)
Chloroflexi	4	1
Spirochaetes	12	5

Widespread in all proteobacterial classes and many other non-proteobacterial groups
